# Hormone replacement therapy and the risk of asthma attacks: population-based cohort study

**DOI:** 10.1183/23120541.01599-2025

**Published:** 2026-08-10

**Authors:** Bohee Lee, Haresh Anpalagan, Ernie Wong, Tricia Tan, Chloe I. Bloom

**Affiliations:** 1 National Heart and Lung Institute, Imperial College London, London, UK; 2 Department of Metabolism, Digestion and Reproduction, Faculty of Medicine, Imperial College London, London, UK

## Abstract

**Background:**

Women experience higher rates and greater severity of asthma after puberty, suggesting that sex hormones play a key role in airway disease. Hormone replacement therapy (HRT), which acutely alters circulating sex hormone levels, provides a useful model to examine their effects on asthma attacks. We sought to examine the association between HRT use and asthma attacks.

**Methods:**

We obtained a cohort of women with asthma, aged 45–60 years, using nationwide UK primary care health records linked to hospital and mortality data, 2004–2020, to compare HRT users to nonusers. We applied inverse-probability of treatment weighting and Cox proportional hazards, accounting for demographics, asthma severity and comorbidities; stratifying by potential modifiers, including body mass index (BMI), blood eosinophil count, smoking and HRT type (oestrogen-only, progesterone-only and combined).

**Results:**

182 010 women were eligible for the study, of whom 9663 were incident HRT users and 172 347 were nonusers. The median age was 52 years (interquartile range 50–55 years). HRT users and nonusers were broadly similar in terms of BMI and smoking history, but nonusers had slightly higher proportions residing in more deprived areas, with more comorbidities, higher reliever use and asthma attacks in the year before study entry, but lower use of preventer inhalers. After applying weighting, there was no association found between HRT use and asthma attacks (weighted hazard ratio 0.97, 95% CI 0.90–1.04). There was no modification of that association by blood eosinophil level, BMI, smoking history or HRT type.

**Conclusion:**

HRT use is not associated with asthma attacks in women with established asthma aged 45–60 years.

## Shareable abstract

**Hormone replacement therapy use is not associated with asthma attacks in women with established asthma aged 45–60 years** https://bit.ly/40zk6px

## Introduction

Sex differences play a significant role in the prevalence, severity and progression of asthma [1]. Women are more likely than men to experience adult-onset asthma and asthma attacks, and hormonal fluctuations across the menstrual cycle, pregnancy and menopause appear to influence symptom control. Understanding the interaction between sex hormones and asthma is therefore essential for optimising asthma care. Endogenous hormone levels fluctuate substantially during puberty, pregnancy and menopause, and exogenous hormones are most prescribed to women as the oral contraceptive pill (OCP) or hormone replacement therapy (HRT), providing an opportunity to study their effects.

Mechanistic studies investigating the relationship between sex hormones and asthma have found mixed results; oestrogen and progesterone have been linked to both promoting and mitigating airway inflammation, bronchial hyperresponsiveness and airway remodelling [2]. All but one HRT observational study have centred on asthma onset as the outcome [3–5]. However, because symptom onset typically precedes healthcare presentation and diagnostic confirmation, and the recorded diagnosis date may lag true onset in routine data, studies of incident asthma are vulnerable to misclassification of onset and potential reverse causation. Observational designs that clearly define when exposure and outcomes occur, such as an asthma attack, are less susceptible to these biases. We previously examined the association between asthma attacks and OCP use, and separately asthma attacks and pregnancy [6, 7]. Our previous findings suggest heterogeneity by hormonal formulation and patient phenotype. Progesterone-only oral contraceptives were associated with higher asthma attack risk, particularly in younger women and those with type-2 inflammation or lower corticosteroid use, whereas combined oral contraceptives were not. In pregnancy, we observed fewer exacerbations managed in primary care but more unscheduled hospital visits, consistent with changes in healthcare-seeking behaviour, prescribing thresholds or clinical pathways during pregnancy that may redistribute events across care settings. These studies included younger women than typical HRT users who may have different underlying asthma pathophysiology and were assessing different levels of sex hormones.

To date, only one study has investigated the relationship between HRT and asthma attacks [8]. Using electronic health records, the authors reported an association between HRT and attacks; however, this association was limited to women with a history of HRT use, no association was observed among current HRT users. This pattern lacks a clear mechanistic explanation and may reflect design-related bias, including prevalent-user bias and residual confounding, because the study did not adopt a new-user (incident-user) design. The new-user approach helps avoid prevalent-user bias, reduces confounding by indication, strengthens temporality, limits reverse causation, captures early effects at treatment initiation and minimises immortal time bias by defining cohort entry at the index date and assigning exposed person-time only from incident HRT initiation [9].

Here, we applied a new-user design with 2 years’ follow-up to evaluate the association between HRT and asthma attacks. We also assessed potential effect modification by clinically relevant factors, including HRT type (combined, oestrogen-only and progesterone-only), body mass index (BMI), smoking history and blood eosinophil count, to explore heterogeneity across asthma endotypes.

## Methods

### Data source

We used the Clinical Practice Research Datalink, Aurum, a nationally representative longitudinal dataset covering approximately 20% of the UK. The dataset contains primary care medical records, including data on diagnoses, symptoms and medications, individually linked to Hospital Episode Statistics (English hospital admission and emergency department data) and Office for National Statistics death registration data.

### Study population and study design

Our study population was women aged 45–60 years with a recorded diagnosis of asthma. Asthma was defined as patients who had at least two asthma codes prior to entering the cohort and at least one asthma inhaler prescription in the year before cohort entry (data available at https://github.com/BoheeLEE/SevereAsthma). The start of the asthma cohort was defined as the latest date of 1 January 2004, date of registration with their general practice, their 45th birthday or their first asthma code.

We used a new-user study design, therefore patients using HRT before cohort entry (defined as any HRT prescription at any time-point before cohort entry in the patient's general practitioner (GP) records) were excluded. HRT users were those women who started using HRT during follow-up and were prescribed at least two consecutive HRT prescriptions (defined as within 60 days). Women with intermittent HRT use were excluded. HRT users were censored 31 days after their last HRT prescription. HRT may be prescribed as or as combined oestrogen+progestogen therapy if the uterus is intact (to protect the endometrium) or as oestrogen-only if the uterus has been removed. Progesterone-only HRT is prescribed in women who cannot take oestrogen, such as those with a history of oestrogen-sensitive breast cancer, high risk of thrombosis or migraine with aura, and in those needing endometrial protection while using local vaginal oestrogen or a levonorgestrel-releasing intrauterine system.

Nonusers were women not prescribed HRT during the cohort follow-up period. The study start date was therefore the first date of HRT prescription for the HRT users and the cohort entry date for the nonusers. The study follow-up ended at the earliest time of the following: 24 months after study start, 31 December 2020, end of their general practice registration date, date of death, 60th birthday or change between exposure group (a nonuser starting HRT or an HRT user stopping HRT).

### Outcome and covariates

The outcome was an asthma attack, defined as a short course of oral corticosteroids, asthma-related accident and emergency visit, hospital admission or death (International Classification of Diseases-10 codes J45 and J46); asthma attacks occurring within a 14-day period were considered as one event [10].

Potential confounders or major risk factors were selected *a priori* and visualised in a directed acyclic graph (supplementary figure E1). These included age, socioeconomic deprivation (defined using the quintiles of Index of Multiple Deprivation, 1 being the least deprived), GP practice, BMI (categorised as underweight: <18.5 kg·m^–2^; normal: 18.5–24.9 kg·m^–2^; overweight: 25.0–29.9 kg·m^–2^; and obese: ≥30.0 kg·m^–2^), smoking history (never-smoker, ex-smoker and current smoker), inhaled asthma medication in the 12 months before the study start date (categorised as reliever only, infrequent (<4 prescriptions) inhaled corticosteroids (ICS) only, regular (≥4 prescriptions) ICS only, infrequent ICS with add-on medication including a long-acting bronchodilator or leukotriene receptor antagonist, and regular ICS with add-on medication), short-acting beta-agonist (SABA) frequency, blood eosinophil counts and asthma attack in the 12 months before the study start date (categorised as none, one and >1), frequency of attending GP practice for clinical visits, comorbidities including atopy, gastro-oesophageal reflux (GOR), obstructive sleep apnoea (OSA), depression, anxiety, COPD), hypertension and cardiovascular disease (CVD) defined using appropriate codes (data available at https://github.com/BoheeLEE).

### Statistical analysis

To determine the association between HRT use and asthma attacks, we applied propensity score methods. To achieve exchangeability (symmetry in the probability distribution between HRT users and nonusers) conditional on the available covariates, we used stabilised inverse probability treatment weighting (IPTW). Weights were estimated using probability of HRT assignment derived from multivariable logistic regression models accounting for the covariates listed above. The missing indicator approach was used to deal with missing data. Absolute standardised differences were calculated to assess covariate balance, with a difference <0.10 indicating good balance. The unadjusted cumulative incidence of first asthma attacks was visualised using a Kaplan–Meier plot. Weighted Cox proportional hazards models were fitted to estimate the relative risk. The proportional hazards assumption was graphically satisfied. To investigate effect modification, we stratified the models by the following binary variables: type of HRT (oestrogen-only or combined; there were insufficient progesterone-only users), BMI (<25 or ≥25kg·m^−2^), smoking (never or ex-/current), blood eosinophil count (<0.3 or ≥0.3×10^9^·L^−1^). Data handling and analysis was carried out using STATA software version 17 (StataCorp, College Station, Texas, USA).

## Results

### Patient characteristics

There were 182 010 women with asthma meeting eligibility criteria to enter the cohort, of whom 9663 were HRT users and 172 347 were nonusers (figure E2). Of the HRT users, 52% (n=5025) were prescribed combined HRT, 46% (n=4446) were prescribed oestrogen-only HRT and 2% (n=193) were prescribed progesterone-only HRT.

The median age of HRT users was 52 years (interquartile range 50–55 years), just over half were overweight or obese, around one-fifth were current smokers, just under half had documented anxiety or depression, one-tenth had documented GOR and nearly 40% had atopy (table 1). Nonusers were similar in these parameters. However, nonusers had a slightly higher proportion residing in more deprived areas, more OSA, COPD, type-2 diabetes, hypertension and CVD.

**TABLE 1:** Baseline characteristics of the patients before and after propensity score weighting

	Before weighting					After weighting				
	HRT		No HRT			HRT		No HRT		
	N	%	N	%	SMD	N	%	N	%	SMD
**Total**	9663	100	172 347	100		9663	100	172 347	100	
**Age (years: median, IQR)**	52 (50–55)		52 (49–57)		0.014	52 (50–56)		52 (50–56)		−0.049
**BMI**										
Normal	2628	27.2	37 399	21.7	0.128	2136	22.1	37 916	22.0	0.002
Underweight	106	1.1	2241	1.3	−0.024	116	1.2	2241	1.3	−0.006
Overweight	3305	34.2	57 219	33.2	0.022	3189	33.0	57 219	33.2	−0.004
Obese	2184	22.6	49 808	28.9	−0.145	2812	29.1	49 291	28.6	0.011
Missing	1449	15.0	25 680	14.9	0.001	1411	14.6	25 680	14.9	−0.009
**Smoking**										
Never	3682	38.1	58 081	33.7	0.093	3189	33.0	58 426	33.9	−0.019
Current	2068	21.4	44 466	25.8	−0.104	2541	26.3	44 121	25.6	0.018
Ex-smoker	3527	36.5	64 285	37.3	−0.017	3566	36.9	64 285	37.3	−0.006
Missing	387	4.0	5515	3.2	0.043	358	3.7	5687	3.3	0.024
**Socioeconomic deprivation (IMD)**										
1 (least deprived)	2261	23.4	31 712	18.4	0.123	1788	18.5	32 057	18.6	−0.004
2	2145	22.2	33 263	19.3	0.073	1846	19.1	33 435	19.4	−0.008
3	1923	19.9	32 574	18.9	0.024	1855	19.2	32 746	19.0	0.005
4	1681	17.4	35 503	20.6	−0.080	1981	20.5	35 159	20.4	0.001
5	1652	17.1	39 467	22.9	−0.145	2203	22.8	38 778	22.5	0.005
**GP consultation (quartiles)**										
1 (least frequent)	2570	26.6	43 604	25.3	0.028	2493	25.8	43 776	25.4	0.010
2	2483	25.7	43 087	25.0	0.016	2319	24.0	43 087	25.0	−0.024
3	2464	25.5	42 742	24.8	0.018	2319	24.0	42 742	24.8	−0.020
4	2145	22.2	42 914	24.9	−0.064	2532	26.2	42 742	24.8	0.033
**Comorbidities**										
Atopy	3856	39.9	63 079	36.6	0.068	3662	37.9	63 251	36.7	0.024
GORD	1102	11.4	22 233	12.9	−0.046	1314	13.6	22 060	12.8	0.023
OSA	39	0.4	1034	0.6	−0.017	48	0.5	862	0.5	−0.011
Anxiety	1826	18.9	32 746	19.0	−0.002	1894	19.6	32 746	19.0	0.014
Depression	2744	28.4	45 500	26.4	0.046	2648	27.4	45 672	26.5	0.020
COPD	377	3.9	14 822	8.6	−0.195	850	8.8	14 305	8.3	0.018
T2DM	570	5.9	16 545	9.6	−0.142	1015	10.5	16 201	9.4	0.036
Hypertension	1121	11.6	33 263	19.3	−0.215	1894	19.6	32 574	18.9	0.018
CVD	271	2.8	8617	5.0	−0.114	493	5.1	8445	4.9	0.007
**Asthma variables**										
SABA frequency (past year)										
0–2	1614	16.7	54 979	31.9	−0.359	2967	30.7	53 600	31.1	−0.007
3–8	2541	26.3	47 568	27.6	−0.030	2648	27.4	47 395	27.5	−0.003
≥9	5508	57.0	69 801	40.5	0.334	4039	41.8	71 352	41.4	0.009
Asthma therapy (past year)										
SABA only	879	9.1	27 231	15.8	−0.206	1517	15.7	26 714	15.5	0.005
ICS only infrequent	1305	13.5	41 191	23.9	−0.269	2203	22.8	40 157	23.3	−0.012
ICS only regular	2802	29.0	42 397	24.6	0.099	2416	25.0	42 742	24.8	0.004
ICS+LABA/LTRA infrequent	309	3.2	12 237	7.1	−0.176	628	6.5	11 720	6.8	−0.013
ICS+LABA/LTRA regular	4377	45.3	49 464	28.7	0.350	2899	30.0	50 842	29.5	0.010
Blood eosinophils										
<300 cells·µL^−1^	3276	33.9	58 253	33.8	0.002	3218	33.3	58 253	33.8	−0.011
≥300 cells·µL^−1^	2377	24.6	44 810	26.0	−0.032	2580	26.7	44 638	25.9	0.017
Missing	4010	41.5	69 283	40.2	0.026	3875	40.1	69 456	40.3	−0.004
Exacerbations (past year)										
None	7653	79.2	131 156	76.1	0.074	7412	76.7	131 501	76.3	0.010
1	1092	11.3	23 612	13.7	−0.071	1276	13.2	23 267	13.5	−0.010
>1	918	9.5	17 579	10.2	−0.024	976	10.1	17 579	10.2	−0.003

BMI: body mass index; CVD: cardiovascular disease; GORD: gastro–oesophageal reflux disease; GP: general practitioner; HRT: hormone replacement therapy; ICS: inhaled corticosteroid; IMD: Index of Multiple Deprivation; IQR: interquartile range; LABA: long-acting beta-agonist; LTRA: leukotriene receptor antagonist; OSA: obstructive sleep apnoea; SABA: short-acting beta-agonist; T2DM: type-2 diabetes mellitus; SMD: standardised mean difference.

In terms of their asthma, HRT users had a higher frequency of SABA use and were more likely to use higher doses of preventer medication (table 1). However, 21% of HRT users had an asthma attack in the year before the study, as compared to 24% of nonusers. The proportions with elevated blood eosinophil count and with no recorded blood count were similar.

After weighting, the distribution of variables between HRT users and nonusers were balanced (standardised mean difference <0.1) (table 1).

### Association between HRT and asthma attacks

**FIGURE 1:**
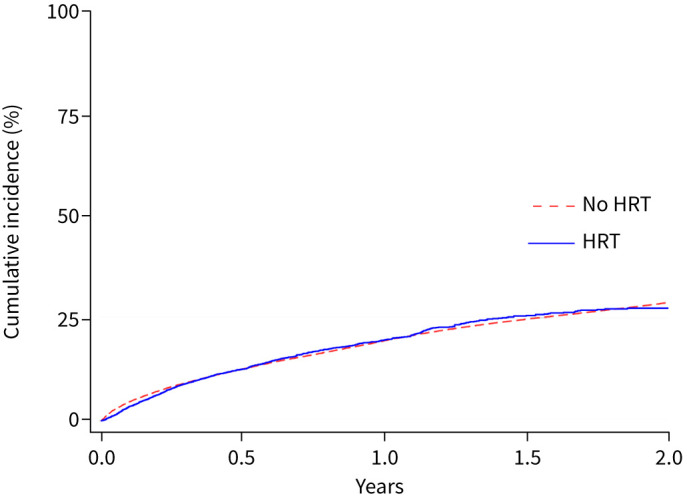
Cumulative, unadjusted, incidence of asthma attacks by hormone replacement therapy (HRT) use.

The incidence rates of first asthma attack per 10 person-years were 2.43 (95% CI 2.30–2.58) in HRT users and 1.80 (95% CI 1.78–1.28) in nonusers (figure 1). In the combined HRT users the incidence was 2.34 (95% CI 2.17–2.52) and in the oestrogen only HRT users it was 2.42 (95% CI 2.31–2.74).

**FIGURE 2:**
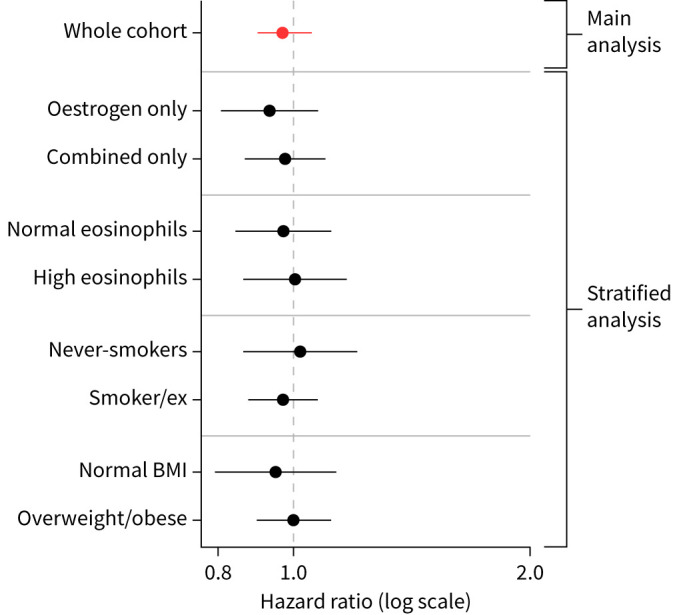
Association between hormone replacement therapy use and asthma attacks (main analysis) and any modification of that association by potential covariates (stratified analysis). Inverse probability treatment weighted Cox proportional hazards. BMI: body mass index.

After accounting for confounding, using IPTW, there was no association between HRT use and asthma attacks (weighted hazard ratio 0.97, 95% CI 0.90–1.04) (figure 2).

### Possible modifiers of HRT and asthma attack association

The models were stratified by the following potential effect modifiers: HRT-type, blood eosinophils, BMI and smoking. There was no significant modification of the association between HRT and asthma attacks by any of the variables (figure 2 and table E1).

## Discussion

In this large population-based cohort, we found no significant association between HRT use and asthma attacks, including with different HRT compounds and asthma phenotypes. Our findings parallel those from the only previous study of HRT use and asthma attacks [8]. Although the earlier study concluded HRT increased the risk of asthma attacks, this was only found in those women with a past history of using HRT, not in women currently using HRT. Yet the biological rationale for these findings has not yet been established and prevalent-user bias cannot be ruled out.

Our present findings also align with our study investigating the association of the OCP and asthma attacks [6]. In the OCP study, we did not find an association between asthma attacks and OCP, except with women using the progesterone-only OCP who were aged under 35 years old. Progesterone levels are high up until the age of reproductivity, falling substantially around the mid to late thirties [11, 12]. Our OCP findings could therefore be explained by combined high endogenous and exogenous progestogen levels in younger women. Due to the low use of progesterone-only HRT, we were unable to investigate any specific progesterone-related effect in this present study of older women.

Several studies have investigated the use of HRT and the development of asthma. Three studies from the 1990s reported a significant increase in new-onset asthma, one found higher risks in obese individuals but two found higher risks in nonobese individuals [3, 13, 14]. In contrast, a UK study found a reduced risk of new-onset asthma and no effect from obesity [4]. A Danish health records study found an increase in asthma incidence but used ICS, risking misclassification [5]. These contradictory findings could be explained by certain modifiers not yet addressed, such as asthma endotype; however, they may also have occurred due to biases related to the study design, such as misclassification of new-onset asthma and reverse causation.

This study has some strengths. Including the use of a large, nationwide dataset and use of a new-user design with clearly defined exposure and outcome windows, reducing prevalent-user bias and reverse causation, and strengthening causal interpretation. This study also has some limitations. As with all observational studies there is a risk of residual confounding. We were not able to determine adherence to prescriptions, including HRT and asthma medication. We did not know the symptoms that led women to use HRT, which could have biased findings. In the UK, although some vaginal HRT can be purchased over the counter directly from pharmacists, all systemic HRT can only be acquired through a prescription. We were not able to investigate progesterone-only HRT due to its low use, nor investigate the effects of the HRT-delivery route. Asthma may have been misdiagnosed as patients were not necessarily under the care of respiratory specialists, but all women had to have at least two asthma codes and be prescribed a reliever or preventer in the year prior to study entry. Some asthma markers were not available, including lung function data, and blood eosinophil count was not available for all women. We also did not have information on ethnicity.

### Conclusion

In this large, population-based, new-user cohort of women aged 45–60 years with asthma, HRT use was not associated with increased asthma attack risk and results were consistent across HRT regimens and clinical strata. These data support shared decision-making that prioritises menopausal symptom relief and standard safety considerations, alongside routine asthma monitoring. Synthesising findings across our HRT, OCP and pregnancy cohorts, the influence of sex hormones on asthma appears most pronounced in younger women, potentially linked to progesterone.

## Data Availability

This study utilised anonymised electronic health records, Clinical Practice Research Datalink (CPRD) obtained under license from the UK Medicines and Healthcare Products Regulatory Agency. According to the UK Data Protection Act, electronic health records are regarded as “sensitive data”, which prevents data sharing *via* public deposition. To access CPRD data and linked data, such as Hospital Episodes Statistics, data from the Office for National Statistics and multiple deprivation index data, requires approval *via* CPRD's Research Data Governance Process (https://cprd.com/data-access). Data management was provided by the Big Data and Analytical Unit at the Institute of Global Health Innovation.

## Supplementary material

**Please note:** supplementary material is not edited by the Editorial Office, and is uploaded as it has been supplied by the author. Supplementary material 01599-2025.SUPPLEMENT
