# Supplementary material for: Hormone replacement therapy and the risk of asthma attacks: population-based cohort study
Source: ERJ Open Res. 2026 Aug 10;12(4):01599-2025. doi: 10.1183/23120541.01599-2025 (PMC13454878; doi:10.1183/23120541.01599-2025)
Supplement: Supplementary file 1 [file 01599-2025.SUPPLEMENT.pdf]

Online Repository

Figure E1. Directed acyclic graph visualising potential confounders and major risk factors

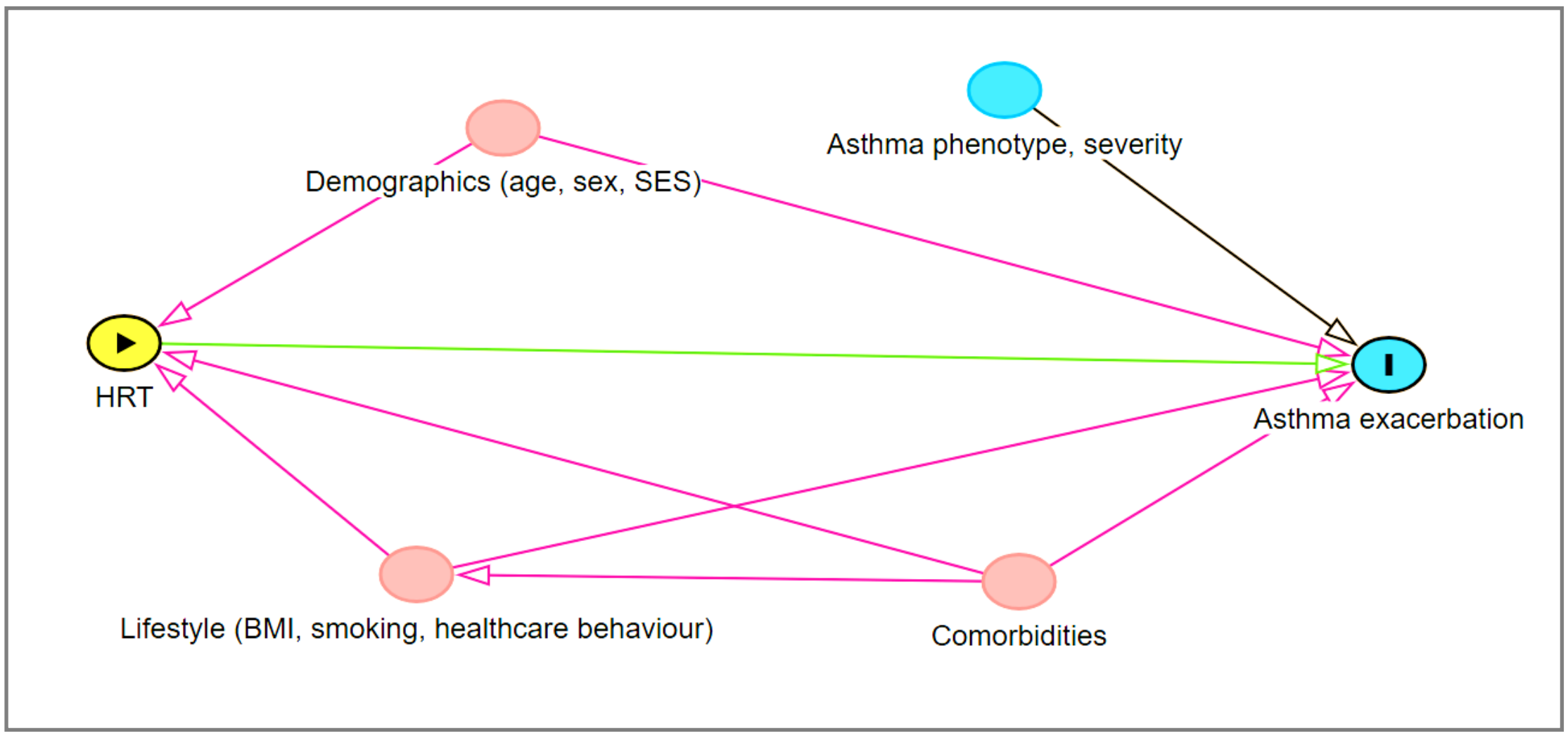

Figure E2. Flow diagram of the study

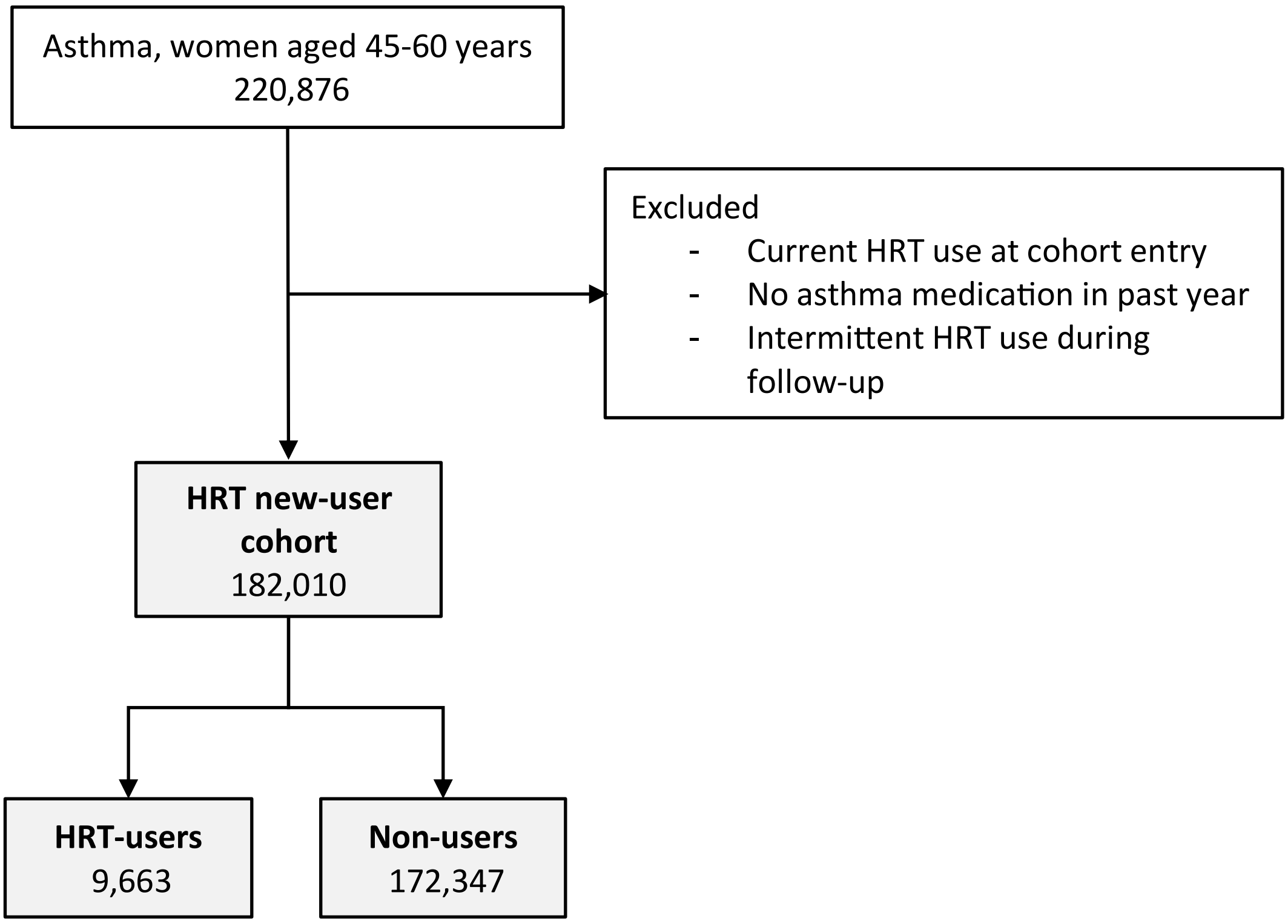

Table E1. Stratified analysis

Variable	Strata	Weighted-HR	p-value	95% CI
**HRT-type**	**Oestrogen-only**	0.93	0.287	0.81-1.07
	**Combined**	0.97	0.652	0.87-1.10
**Blood eosinophils**	**High**	1.00	0.962	0.88-1.16
	**Low**	0.97	0.638	0.84-1.11
**Smoking**	**Never**	1.02	0.818	0.86-1.20
	**Ex/current**	0.97	0.529	0.87-1.07
**BMI**	**Normal**	0.94	0.507	0.79-1.21
	**Overweight/obese**	1.00	0.999	0.89-1.11
